# Protein mistranslation protects bacteria against oxidative stress

**DOI:** 10.1093/nar/gku1404

**Published:** 2015-01-10

**Authors:** Yongqiang Fan, Jiang Wu, Matthew H. Ung, Nicholas De Lay, Chao Cheng, Jiqiang Ling

**Affiliations:** 1 Department of Microbiology and Molecular Genetics, Medical School, University of Texas Health Science Center, Houston, TX 77030, USA; 2 Department of Genetics, Geisel School of Medicine at Dartmouth, Hanover, NH 03755, USA; 3 Graduate School of Biomedical Sciences, Houston, TX 77030, USA

## Abstract

Accurate flow of genetic information from DNA to protein requires faithful translation. An increased level of translational errors (mistranslation) has therefore been widely considered harmful to cells. Here we demonstrate that surprisingly, moderate levels of mistranslation indeed increase tolerance to oxidative stress in *Escherichia coli*. Our RNA sequencing analyses revealed that two antioxidant genes *katE* and *osmC*, both controlled by the general stress response activator RpoS, were upregulated by a ribosomal error-prone mutation. Mistranslation-induced tolerance to hydrogen peroxide required *rpoS, katE* and *osmC*. We further show that both translational and post-translational regulation of RpoS contribute to peroxide tolerance in the error-prone strain, and a small RNA DsrA, which controls translation of RpoS, is critical for the improved tolerance to oxidative stress through mistranslation. Our work thus challenges the prevailing view that mistranslation is always detrimental, and provides a mechanism by which mistranslation benefits bacteria under stress conditions.

## INTRODUCTION

**Figure 1.**
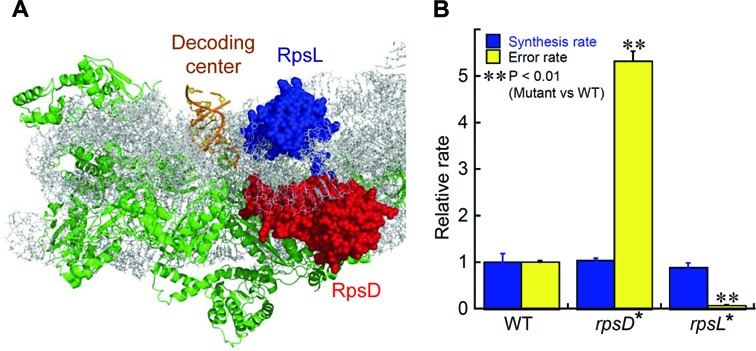
Mutations in ribosomal proteins alter translational fidelity. (**A**) Crystal structure of the 30S ribosome (PDB: 1IBL) (15). RpsD and RpsL are close to the decoding center of the ribosome, where the anticodon of tRNA matches the codon on the mRNA. (**B**) Mutation I199N in *E. coli* RpsD increased read-through of an in-frame UAG stop codon in *lacZ*, and mutation K42N in RpsL decreased the mistranslation rate (shown in yellow). The relative protein synthesis rates, measured with an YFP reporter, are shown in blue. The error bars represent standard deviations (*n* ≥ 3).

Protein synthesis is a central process in all three domains of life, and translational fidelity is carefully controlled in cells (1,2). The average translational error rate is around 10^−4^-10^−3^ per codon (3,4). Maintaining such translational fidelity requires correct pairing of amino acids and tRNAs by aminoacyl-tRNA synthetases (aaRSs), and faithful decoding of mRNA codons by the corresponding aminoacyl-tRNAs (aa-tRNAs) on the ribosome. In addition to the initial selection of cognate substrates, many aaRSs and the ribosome use proofreading mechanisms to correct mistakes and ensure translational fidelity (5–9). AaRSs occasionally misactivate amino acids that are structurally similar to the cognate ones, and use an editing function to hydrolyze misactivated amino acids and misacylated tRNAs (1,10). Mutations that cause editing defects in aaRSs lead to mistranslation of specific codons. For example, an editing defect in phenylalanyl-tRNA synthetase results in Tyr misincorporation at Phe codons (11). Selection of the correct tRNA by the aaRS is typically an accurate process (12), but misacylation of non-cognate tRNAs may increase under viral infection or oxidative stress conditions (13). The ribosome also uses kinetic proofreading to distinguish between cognate and near-cognate aa-tRNAs (8). Binding of cognate (but not near-cognate) aa-tRNAs induces a conformational change (domain closure) at the decoding center of the ribosome (14,15) and promotes the forward reaction of dipeptide-bond formation (16). Disruption of ribosomal proofreading increases global translational errors. For example, aminoglycoside antibiotics increase mistranslation by inducing domain closure in the ribosome (17–19). Mutations in the ribosomal proteins RpsD, RpsE and 16S ribosomal RNA have also been shown to increase errors during translational initiation, elongation and termination (12,20–22). It is suggested that the ribosomal ambiguous mutations in RpsD (Figure 1) and RpsE stabilize the closed form of the ribosome, therefore allowing erroneous decoding by near-cognate tRNAs (12). On the other hand, mutations in RpsL increase the energy barrier for domain closure in the presence of near-cognate tRNAs and decrease the level of translational errors (12).

It has been proposed that maintaining translational fidelity poses a dominant constraint on coding-sequence evolution, suggesting that loss of fitness is widely associated with increased mistranslation (23). Indeed, mistranslation has been shown to cause growth defects in bacteria (24,25), mitochondrial dysfunction in yeast (26), apoptosis in mammalian cells (27) and neurodegeneration in mice (28). It is therefore commonly thought that mistranslation is harmful to the cell. Growing evidence suggests that mistranslation is far more wide-spread than previously recognized. It has been shown that ribosomes purified from different natural *Escherichia coli* isolates vary over 10-fold in mistranslation rates (29), implying that various levels of translational fidelity are favored under distinct environments. Several aaRSs from mycoplasma and yeast mitochondria have lost the editing function conserved in other species and compartments, therefore fail to maintain fidelity during aa-tRNA synthesis (30–32). It has been suggested that high translational error rates in mycoplasma may benefit the microorganism by causing phenotypic diversity with a statistical proteome (30). Translational fidelity is also found to be regulated under stress conditions. In mammalian cells, viral infection and oxidative stress enhance misincorporation of Met into the proteome, which is suggested to protect the proteome from oxidative damage (13). In bacteria, nutrient starvation substantially increases read-through of stop codons (33), and oxidative stress leads to Ser misincorporation at Thr codons through impairing the editing function of threonyl-tRNA synthetase (34,35). These observations have prompted recent discussion that mistranslation could be adaptive and beneficial under certain conditions (36,37), but experimental evidence is further needed to support this concept, and very little is known about the underlying mechanisms. Here we combined systems biology, genome engineering, genetics and microbiology tools to demonstrate that mistranslation resulting from a ribosomal mutation or misreading of Arg codons substantially enhanced tolerance to oxidative stress triggered by hydrogen peroxide (H_2_O_2_) in *E. coli*. We further found that protection against H_2_O_2_ by mistranslation depended on activation of the general stress response controlled by the sigma factor RpoS. An elevated level of RpoS, which required a small RNA DsrA, increased the mRNA levels of a catalase (KatE) and a peroxidase (OsmC), which contributed to protection against H_2_O_2_ under mistranslation conditions.

## MATERIALS AND METHODS

### Strains, plasmids, growth conditions and reagents

The plasmids used for overexpression of candidate genes, *katE, osmC* and *rpoS*, were from the ASKA *E. coli* open reading frame (ORF) library (38). Plasmids for overexpressing small regulatory RNAs (sRNAs) were from the sRNA overexpression library (39). pZS*11 was provided by Drs Arvind R. Subramaniama and Philippe Cluzela (Harvard University). pKD46 was from the *E. coli* Genetic Stock Center (Yale University). *E. coli* cells were grown in Luria broth (LB) or on Luria agar plates. Antibiotics were used at the following concentrations: ampicillin (Amp), 100 μg/ml; chloramphenicol (Chl), 25 μg/ml. Arabinose and Isopropyl-β-D-thiogalactopyranoside (IPTG) were used at a final concentration of 10 mM and 100 μM, respectively. Stable hydrogen peroxide (H_2_O_2_) stock solution (10 M) was obtained from Fisher (Fair Lawn, NJ, USA), and fresh dilutions were prepared immediately before use. Antibiotics and chemicals were purchased from Sigma-Aldrich (St. Louis, MO, USA). Antibody against RpoS was from Santa Cruz Biotechnology, Inc (Dallas, TX, USA). RNase-free DNase I and o-Nitrophenyl-β-D-galactopyranoside (ONPG) were from Thermo (Rockford, IL, USA).

### Genome engineering of bacterial strains

All strains used in this study were derivatives of *E. coli* K-12 strain MG1655 (F-, *λ^−^, rph-1*), which was obtained from The *E. coli* Genetic Stock Center at Yale University. rpsD* and rpsL* mutants were generated with a modified multiplex automated genome engineering (MAGE) method (40,41). Instead of using genome-integrated lambda-Red recombinase in the original publication, we used arabinose-induced lambda-Red recombinase expressed from plasmid pKD46 [*repA101ts*, Amp^R^, *P_araB_*] (42). The plasmid was removed after mutations were introduced into the genome by incubation of cells at a non-permissive temperature (42°C). The mutations were confirmed by both polymerase chain reaction (PCR) and Sanger sequencing. All in-frame gene-deletion mutants were constructed as described using chloramphenicol as the resistance marker (42). All the mutants were verified by PCR, and the antibiotic resistance genes were subsequently removed from the deletion strains using plasmid pCP20 (42). pCP20 carries a temperature-sensitive origin, and was cured at 43°C. The marker-free deletion mutants were verified by both loss of resistance and PCR.

### Determination of mistranslation and protein synthesis rates

To test the mistranslation rate, a plasmid (pLacZ) harboring a mutant version of *lacZ* with an amber nonsense codon at amino acid position three was used in the β-galactosidase assay as described (43). Based on the Miller units for the enzyme activity, the relative suppression rates in the mutant strains were calculated as a percentage of that in wild-type (WT) strain.

To determine the protein synthesis rate, we used a low-copy number plasmid – pZS*11 [SC101* *ori*, Amp^R^, constitute *P_LtetO-1_* promoter] harboring a yellow fluorescent protein (YFP) gene (*yfp*) variant. Overnight cultures of each strain were diluted 50-fold into 100 μl fresh LB medium containing Amp in 96-well plates. The growth and fluorescence were continuously monitored in a plate reader for at least 12 h. The protein synthesis rates were calculated as described (44).

### RNA sequencing and data analysis

The WT, rpsD* and rpsL* strains were grown at 37°C to mid-log phase in LB medium and harvested for RNA extraction. Total RNA was prepared using the RNAprotect Bacteria Reagent and RNeasy Protect Bacteria Kits (Qiagen, Valencia, CA, USA) according to the user manual. Purification of total RNAs included a step to remove small RNAs, therefore the expression levels of small RNAs in our RNAseq results were not accurate. The library preparation and Illumina sequencing were performed by Axeq Technologies, Inc. (Rockville, MD, USA). Two biological replicates of each strain were sequenced. Analysis of the RNA sequencing data was conducted using the Rockhopper open source software package with default parameters (45). Rockhopper's RNA sequencing analysis platform is designed specifically for bacterial genomes. The reference genome, gene annotation and RNA annotation files for *E. coli* K-12 MG1655 were downloaded from the National Center for Biotechnology Information (NCBI) GenBank^®^ data repository. Ninety one to ninety three percent of 101 base-pair single-end reads from all replicates were successfully aligned to the reference genome. An adjusted *P* < 0.01 (Benjamini–Hochberg procedure) was used to identify differentially expressed genes between mutants and WT strains.

### Quantitative reverse transcription-PCR

Mid-log phase of strains grown in LB medium was harvested. Total RNA was extracted using the hot phenol method and residual chromosomal DNA was removed as previously described (46). Reverse transcription and PCR were performed using the iScript cDNA Synthesis Kit and the SsoAdvanced Universal SYBR Green Supermix kit (Bio-Rad, Hercules, CA, USA) according to the manufacturer's instructions. 16S rRNA was used as an internal reference for normalization. The ΔΔ*C_t_* method was used to obtain the fold changes of target genes in the mutant strains compared to those in the WT strain.

### Survival assay

Cells were grown in LB at 37°C, harvested, washed twice using 83 mM phosphate buffer (pH 7.0), diluted to 0.1 OD_600_ in phosphate buffer and treated with 5 mM H_2_O_2_ at 37°C in the dark. At each time point, aliquots were removed, serially diluted in phosphate buffer and spotted onto LB plates. Plates were incubated at 37°C overnight before the colony-forming units were counted.

### Determination of RpoS protein level and translational rate

To determine the protein level of RpoS, cells from specific growth stages were harvested and washed once with phosphate buffer before sonication to lyse the cells. Western blot was performed with standard procedures. To determine the translational level of RpoS, an *rpoS*::*lacZ* translational fusion reporter was introduced into the chromosome of the WT, rpsD* and rpsL* strains using phage P1 *vir* transduction as described (47). The pBAD-*rpoS*-*lacZ* fragment was amplified from TS323 (48). The LacZ activity was determined as in (43).

## RESULTS

### Ribosomal mistranslation upregulates antioxidant genes

To understand the physiological role of mistranslation, we engineered isogenic *E. coli* strains with various translational error rates using a genome editing approach (40). RpsD and RpsL are located near the decoding center of the 30S ribosome (15) (Figure 1A). Mutations in RpsD (such as I199N) have been shown to cause increased translational errors (12,49), while mutations in RpsL (such as K42N) decrease the rate of mistranslation (49,50). We introduced mutations RpsD I199N (referred to as rpsD* hereafter) and RpsL K42N (rpsL*) separately into the genome of the WT *E. coli* strain MG1655 and tested the suppression rates of the resulting strains at an in-frame UAG stop codon of the *lacZ* gene. The RpsD I199N strain exhibited a 5-fold increase in suppression rate, whereas the RpsL K42N mutation decreased the error rate to 6% compared with the WT strain (Figure 1B). Using YFP as a reporter (44), we found that the protein synthesis rate of the reporter was not affected by either the rpsD* or rpsL* mutation (Figure 1B).

Next, we compared the gene expression profiles of the WT, error-prone rpsD* and error-restrictive rpsL* strains using RNA deep sequencing (Supplementary Table S1). Cells were grown to mid-log phase, and total RNA was extracted for deep sequencing. At a cutoff of *P* value < 0.01, 17 genes were found to be upregulated in the rpsD* strain and downregulated in the rpsL* strain compared with the WT (Table 1 and Supplementary Table S1). The majority of these genes are directly regulated by RpoS, a sigma factor controlling the general stress response (51). Interestingly, two antioxidant genes—*katE* and *osmC*—were upregulated in the rpsD* strain. KatE is one of the two catalases in *E. coli* that scavenges H_2_O_2_ (52). Unlike KatG that is induced by H_2_O_2_ through the OxyR response (53) (Supplementary Table S2), KatE is known to be induced by RpoS during stationary phase or in response to stress (54). OsmC protects bacteria against H_2_O_2_ by reducing organic hydroperoxide, and is positively regulated by RpoS (55,56). We further confirmed the increased and reduced expression patterns of *katE* and *osmC* in the rpsD* and rpsL* strains relative to the WT strain, respectively, using quantitative reverse transcription PCR (qRT-PCR) (Supplementary Figure S1A).

**Table 1.** Genes that are upregulated in the error-prone rpsD* strain and downregulated in the error-restrictive rpsL* strain

Name	Product	*P* value rpsD*/WT	Fold rpsD*/WT	*P* value rpsL*/WT	Fold rpsL*/WT	Direct RpoS regulation^a^
*cbdB*	Cytochrome bd-II oxidase, subunit II	1.5E-03	1.8	4.1E-04	0.38	Yes
*fbaB*	Fructose-bisphosphate aldolase class I	1.7E-04	2.5	5.5E-07	0.36	Yes
*hyaA*	Hydrogenase 1, small subunit	3.6E-03	2.0	2.5E-03	0.50	Yes
*hyaC*	Hydrogenase 1, b-type cytochrome subunit	5.5E-04	1.9	7.5E-06	0.33	Yes
*hyaD*	Hydrogenase 1 maturation protease	4.2E-04	2.0	1.7E-04	0.41	Yes
*hyaE*	Putative HyaA chaperone	3.1E-04	2.1	6.2E-04	0.44	Yes
*hyaF*	Protein involved in nickel incorporation into hydrogenase-1 proteins	2.8E-04	2.1	4.8E-04	0.44	Yes
*katE*	Catalase HPII, heme d-containing	1.6E-04	2.7	2.1E-07	0.29	Yes
*narY*	Nitrate reductase 2 (NRZ), beta subunit	3.0E-10	3.2	7.6E-05	0.33	No
*narZ*	Nitrate reductase 2 (NRZ), alpha subunit	3.5E-09	4.0	1.4E-03	0.40	No
*osmC*	Lipoyl-dependent Cys-based peroxidase	1.8E-03	2.1	1.7E-03	0.47	Yes
*osmF*	Putative ABC superfamily transporter	2.9E-04	2.4	1.9E-03	0.46	Yes
*otsB*	Trehalose-6-phosphate phosphatase	4.6E-04	2.1	7.1E-03	0.47	Yes
*phr*	Deoxyribodipyrimidine photolyase	6.4E-03	2.0	2.8E-03	0.48	Yes
*talA*	Transaldolase A	4.5E-03	2.0	1.6E-03	0.47	Yes
*uhpT*	Hexose phosphate transporter	7.9E-08	2.5	1.1E-06	0.00	No
*yohC*	Yip1 family inner membrane protein	1.5E-03	1.8	6.0E-16	0.25	No

^a^This refers to direct binding of RpoS to the promoter according to EcoCyc.

### Ribosomal mistranslation improves tolerance to H_2_O_2_

**Figure 2.**
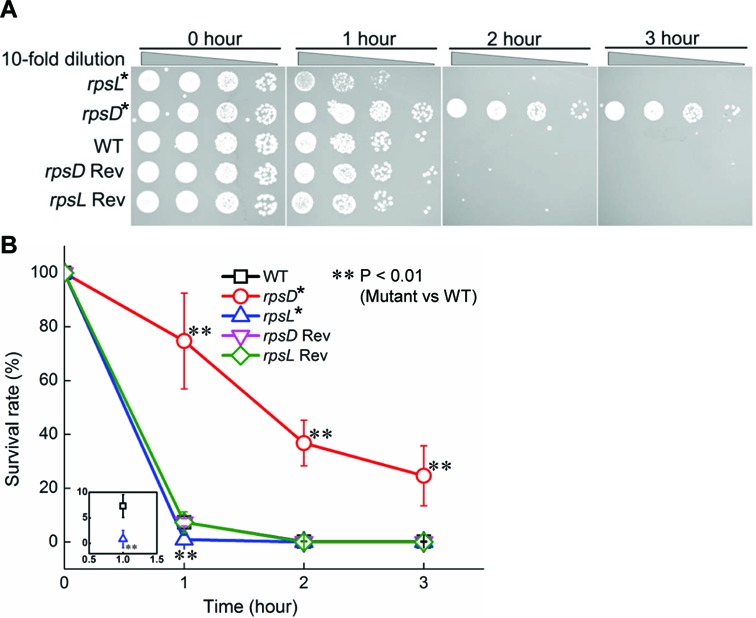
Ribosomal mistranslation protects *E. coli* against H_2_O_2_. (**A**) Survival of *E. coli* strains (mid-log phase) after treatment with 5 mM H_2_O_2_ for various periods of time. The same concentration of H_2_O_2_ has been used throughout this work. (**B**) Quantitation of survival rates. The error bars represent standard deviations (*n* ≥ 3). Rev: revertant.

The observation that *katE* and *osmC* were upregulated in the rpsD* strain prompted us to investigate whether mistranslation would improve bacterial tolerance to oxidative stress. H_2_O_2_ is a reactive oxygen species produced in large amount by macrophages during the host-immune response and by competing microbes in natural environment (52). Treating WT *E. coli* with 5 mM H_2_O_2_ resulted in rapid killing of WT *E. coli* (Figure 2), and controls without H_2_O_2_ exhibited no significant cell death over 3 h (Supplementary Figure S2). The rpsD* mutation significantly increased the survival rate in the presence of H_2_O_2_, whereas the rpsL* mutation sensitized *E. coli* to H_2_O_2_-mediated killing (Figure 2; *P* < 0.01 at 1 h). To validate that the phenotypic changes were not due to non-specific mutations introduced to the genome during strain engineering, we reverted the rpsD* and rpsL* mutations to the WT and tested survival of the resulting strains in the presence of H_2_O_2_. The revertants exhibited the same sensitivity to H_2_O_2_ as the WT strain (Figure 2), confirming that increased translational errors were responsible for the improved tolerance to H_2_O_2_.

### Increased tolerance to H_2_O_2_ in error-prone strain depends on KatE, OsmC and RpoS

**Figure 3.**
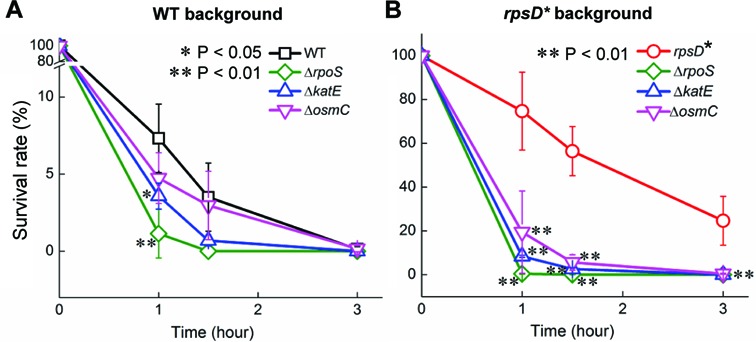
KatE, OsmC and RpoS are required for mistranslation-mediated H_2_O_2_ tolerance. Survival of *E. coli* strains (mid-log phase) after treatment with 5 mM H_2_O_2_ in (**A**) the WT background, and (**B**) the error-prone rpsD* background. The error bars represent standard deviations (*n* ≥ 3).

To address whether upregulation of *katE* and *osmC* contributes to H_2_O_2_ tolerance in the error-prone strain, we knocked out these genes in the WT and rpsD* strains. Deletion of either *katE* or *osmC* significantly increased sensitivity to H_2_O_2_-mediated killing in the rpsD* background (Figure 3), and deleting the master regulator *rpoS* resulted in more severe loss of viability in the presence of H_2_O_2_ in both WT and rpsD* strains. Next, we tested how overexpression of KatE, OsmC and RpoS affected H_2_O_2_ sensitivity (Supplementary Figure S3). Overexpressing KatE or RpoS protected both WT and rpsD* strains against H_2_O_2_, whereas overexpressing *osmC* alone did not improve tolerance to H_2_O_2_. Collectively, these results suggest that both KatE and OsmC are important for detoxifying H_2_O_2_ in the error-prone strain, and KatE possibly plays a more critical role in defense against H_2_O_2_ under mistranslation conditions.

### Mistranslation increases RpoS protein level

**Figure 4.**
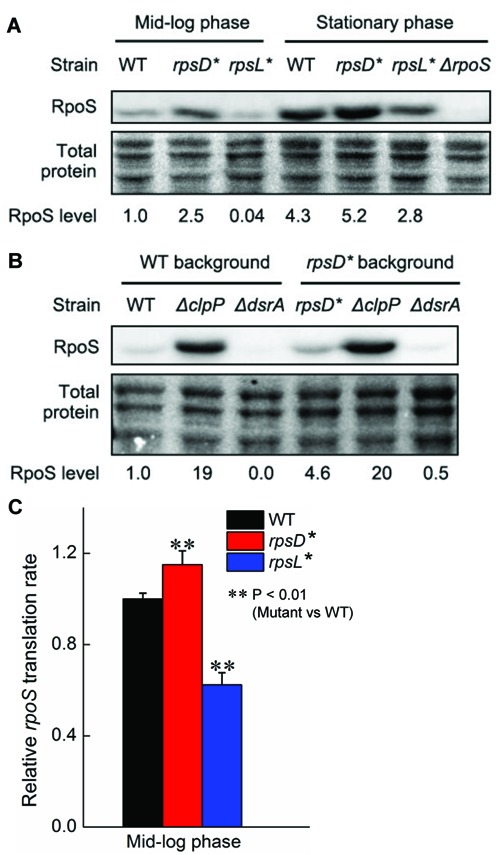
Mistranslation increases RpoS protein level. (**A**) *E. coli* strains were grown to mid-log or stationary phase, and western blot against RpoS (top panel) and Ponceau staining of total proteins (bottom panel) were performed. The error-prone rpsD* mutation increased the RpoS protein level in the mid-log phase. Equal amount of total proteins was loaded in each lane. The figures are representatives of three repeats. (**B**) *E. coli* strains in the WT (MG1655) or rpsD* background were grown to mid-log, and western blot was performed as in (A). Deleting *dsrA* decreased RpoS level, whereas deleting *clpP* stabilized RpoS. (**C**) Translational rate of RpoS determined with a translational fusion of *rpoS-lacZ*, where the mRNA leader sequence of *rpoS* was fused to the *lacZ* gene. The error bars represent standard deviations (*n* ≥ 3).

RNA sequencing and survival assay suggest that the general stress response is activated in the error-prone strain and is critical for the improved tolerance to H_2_O_2_. RpoS is regulated at transcriptional, translational and post-translational levels (51). The mRNA level of RpoS did not significantly change in the rpsD* strain compared to the WT (Supplementary Figure S1B and Supplementary Table S1). To test the RpoS protein level, we used an antibody against RpoS and performed western blot analysis. The RpoS protein level was increased in the rpsD* strain and decreased in the rpsL* strain compared to the WT (Figure 4A). RpoS is degraded by ClpP via an adaptor protein RssB (51,57). It has been suggested that mistranslation increases the level of misfolded proteins, titrating available ClpP and protecting RpoS from degradation (57). In line with this notion, we found that deleting *clpP* significantly increased the protein level of RpoS and tolerance to H_2_O_2_ in the WT strain (Figure 4 and Supplementary Figure S4). Deletion of *clpP* did not further increase survival of the rpsD* strain in the presence of H_2_O_2_, whereas overexpressing ClpP, which likely enhances degradation of RpoS, decreases the tolerance of the rpsD* strain to H_2_O_2_ (Supplementary Figure S4). Deleting *rssB* also increased tolerance to H_2_O_2_ and increased the RpoS protein level in the WT background (Supplementary Figures S4 and S5). While these results support a model that mistranslated proteins titrate ClpP to protect RpoS from degradation, we do not rule out the possibility that a fraction of erroneously translated RpoS becomes resistant to ClpP degradation.

To investigate whether translation of RpoS was affected by mistranslation, we used a *lacZ* fusion with the leader sequence of the *rpoS* gene under the control of an arabinose-inducible promoter. This fusion reporter thus specifically tested translational, but not transcriptional or post-translational regulation of RpoS. Our results revealed that translation of the reporter was slightly but significantly increased in the rpsD* strain (Figure 4C).

### Small RNA DsrA is critical for mistranslation-induced protection against H_2_O_2_

**Figure 5.**
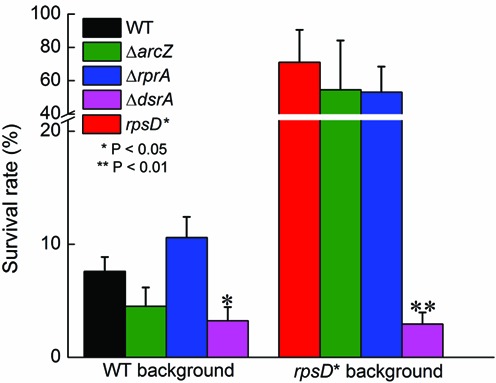
Effects of small RNAs on H_2_O_2_ tolerance in WT and rpsD* strains. DsrA was required for protection against H_2_O_2_ in the error-prone strain. Cells were grown to mid-log phase and treated with 5 mM H_2_O_2_ for 1 h before plating. The error bars represent standard deviations (*n* ≥ 3).

Translation of RpoS is known to be positively regulated by three small RNAs—ArcZ, DsrA and RprA (51,58–60). To test which small RNAs are important for the improved H_2_O_2_ tolerance in the error-prone strain, we deleted each of the three small RNAs from the WT and rpsD* strains. Deleting DsrA significantly decreased the survival rates of the WT and rpsD* strains to the same level, whereas deleting ArcZ or RprA showed no effect on H_2_O_2_ sensitivity (Figure 5). Using qRT-PCR, we verified that DsrA was upregulated 2.5-fold in the rpsD* strain (Supplementary Figure S1B). We further showed that overexpressing DsrA from a plasmid increased tolerance to H_2_O_2_ in the WT and rpsD* background (Supplementary Figure S6). However, in the *rpoS* deletion strains, overexpressing DsrA did not improve the survival of *E. coli* in the presence of H_2_O_2_, suggesting that DsrA protects *E. coli* against H_2_O_2_ via the RpoS response.

### Codon-specific mistranslation also increases tolerance to H_2_O_2_

**Figure 6.**
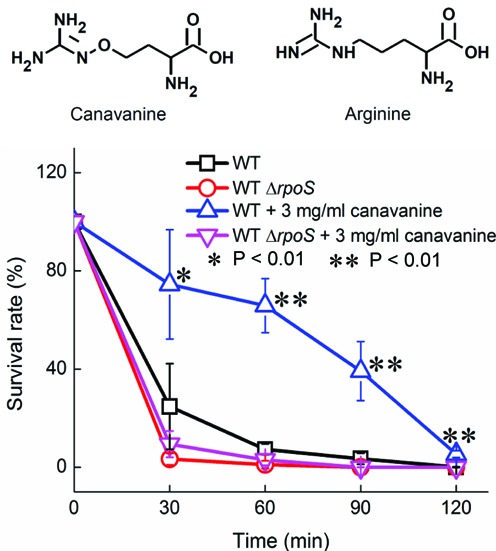
Mistranslation caused by canavanine improves tolerance to H_2_O_2_. The WT and *rpoS-*deletion strains were grown to mid-log phase in the presence and absence of canavanine, and treated with 5 mM H_2_O_2_ before plating. Addition of canavanine protected the WT, but not the *rpoS-*deletion mutant, against H_2_O_2_, suggesting that as the rpsD* mutation, canavanine also increased H_2_O_2_ tolerance in a manner dependent on RpoS. The error bars represent standard deviations (*n* ≥ 3).

To test whether the protective effect of mistranslation against H_2_O_2_ is specific to ribosomal mutations that cause decoding errors at all codons, we introduced codon-specific mistranslation into *E. coli* using canavanine. Canavanine is a structural analog of Arg recognized by the endogenous arginyl-tRNA synthetase (Figure 6), and is misincorporated at Arg codons during protein synthesis (61). Our results revealed that addition of canavanine indeed significantly increased tolerance of WT *E. coli* to H_2_O_2_ (Figure 6). When *rpoS* was deleted, canavanine no longer protected *E. coli* against H_2_O_2_, suggesting that the protective effect against H_2_O_2_ by canavanine also depends on RpoS.

## DISCUSSION

Accumulating evidence has shown that mistranslation is detrimental and even lethal in various organisms and cells (23). For instance, aminoglycoside antibiotics are proposed to kill bacteria by inducing mistranslation and protein misfolding (62), and an editing defect in leucyl-tRNA synthetase increases susceptibility of *E. coli* to near-cognate amino acids (24). A mutation in alanyl-tRNA synthetase that increases misreading of Ala codons selectively damages Purkinje cells and causes neurodegeneration in mice (28), suggesting that different cell types vary in their susceptibility to mistranslation. Even within the same cell, translational errors occurring in different compartments appear to be tolerated at different levels (26). It appears that each type of cell has a threshold to tolerate a certain level of mistranslation, and delicate quality control mechanisms optimize translational fidelity to maximize cellular fitness and maintain selective advantage under normal growth conditions.

In contrast to the common view that mistranslation is toxic, there is recent discussion that mistranslation may be beneficial under stress conditions (36,37). In mammalian cells, misacylation of Met to non-cognate tRNAs is increased upon viral infection or oxidative stress, leading to global misincorporation of Met into non-Met codons (13). It is suggested that an increased level of Met under oxidative stress conditions would protect the proteome from oxidative damage (36). This provides an intriguing model of how mammalian cells adapt to oxidative stress through specific mistranslation of Met. In yeast, it has been shown that serine/leucine ambiguity at CUG codons increases phenotypic diversity and improves tolerance to a number of stresses, including high salt and oxidative stress (63,64). The molecular mechanism leading to such adaptive phenotypes remains to be determined. A recent study reveals that engineered mycobacteria with codon-specific mistranslation increases resistance against rifampicin, an antibiotic targeting the RNA polymerase (65). This is likely because statistically mistranslated RNA polymerase becomes more tolerant to inhibition by rifampicin. The Gram-negative model bacterium *E. coli* is able to tolerate a relatively high level of mistranslation (66). In the present work, we demonstrate that mistranslation resulting from a ribosomal mutation or canavanine protects *E. coli* from H_2_O_2_ through activation of the general stress response. This provides strong experimental evidence to support the notion that mistranslation is adaptive and advantageous under certain stress conditions. We propose that within the threshold of tolerance, mistranslation resulting from stochastic processes or stresses allows cells to produce a statistical proteome and activate adaptive mechanisms. This helps a subpopulation of cells to survive severe stress conditions without introducing permanent genetic changes. Due to the reversibility of stress-induced mistranslation, translational fidelity can be restored upon removal of stresses.

Our results also suggest that mistranslation activates the general stress response by increasing the protein level of RpoS, which is conserved among beta-, gamma- and delta-proteobacteria, including common drug-resistant pathogens *E. coli, Pseudomonas aeruginosa* and *Klebsiella pneumoniae* (67). This suggests that mistranslation-induced tolerance to H_2_O_2_ may be a wide-spread mechanism in Gram-negative bacteria. H_2_O_2_ is massively produced by activated macrophages to kill invading microbes during the host-immune response (68). Mistranslation-induced H_2_O_2_ tolerance may therefore serve as an important mechanism for bacteria to survive the host-immune response. The increased level of RpoS in the error-prone strain results from both translational and post-translational levels of regulation (Figures 4 and 5 and Supplementary Figures S1, S4 and S5). DsrA regulates translation of RpoS by pairing with the mRNA leader sequence to expose the ribosomal binding site (69). DsrA is known to be activated by cold stress, but the activation pathway remains unclear (51,60). We show that mistranslation increases the RNA level of DsrA and translation of RpoS (Figure 4 and Supplementary Figure S1). How DsrA is upregulated in error-prone strains remains to be determined in future studies. Post-translational regulation of RpoS is known to be mediated by the RssB-ClpP system (51). RpoS is specifically recruited to ClpP for degradation by RssB. We show that deleting *clpP* or *rssB* increases tolerance to H_2_O_2_ in the WT *E. coli* strain (Supplementary Figure S4), likely by stabilizing RpoS. In addition to the ribosomal rpsD* mutation, mistranslation of Arg codons by canavanine also increases tolerance to H_2_O_2_ (Figure 6). This suggests that the general stress response is likely to be activated by various types of mistranslation or other stress conditions that lead to protein misfolding.

## CONCLUDING REMARKS

In this work we provide strong experimental evidence that protein mistranslation is adaptive and beneficial under oxidative stress conditions in *E. coli*, and have further clarified the adaptive mechanism. In contrast to previous models that mistranslation provides beneficial effects through production of statistical proteins (36,65), our results suggest that in *E. coli*, mistranslated proteins activate the general stress response and cause adaptation to severe oxidative stress conditions. Mistranslation frequently occurs in natural *E. coli* isolates (29), and is induced by carbon starvation (33), oxidative stress (13,34), aminoglycosides (70) and viral infection (13). Future studies are needed to identify additional environmental factors that cause protein mistranslation, and define the stress responses, adaptive mechanisms and toxicity caused by various types of mistranslation in different organisms.

## SUPPLEMENTARY DATA

Supplementary Data are available at NAR Online.

SUPPLEMENTARY DATA
