# Supplementary material for: Protein mistranslation protects bacteria against oxidative stress
Source: Nucleic Acids Res. 2015 Jan 10;43(3):1740–8. doi: 10.1093/nar/gku1404 (PMC4330365; doi:10.1093/nar/gku1404)
Supplement: SUPPLEMENTARY DATA [file supp_43_3_1740__index.html]

Protein mistranslation protects bacteria against oxidative stress — Protein mistranslation protects bacteria against oxidative stress — SUPPLEMENTARY DATA 

# Protein mistranslation protects bacteria against oxidative stress

## SUPPLEMENTARY DATA

**Files in this Data Supplement:**

- SUPPLEMENTARY DATA
- SUPPLEMENTARY DATA
