# Supplementary material for: Protein mistranslation protects bacteria against oxidative stress
Source: Nucleic Acids Res. 2015 Jan 10;43(3):1740–8. doi: 10.1093/nar/gku1404 (PMC4330365; doi:10.1093/nar/gku1404)
Supplement: SUPPLEMENTARY DATA [file supp_gku1404_nar-03337-r-2014-File008.pdf]

Supplementary Information

Benefits from Errors - Protein Mistranslation Protects Bacteria against Oxidative Stress

Yongqiang Fan[1], Jiang Wu[1], Matthew H. Ung[2], Nicholas De Lay[1,3], Chao Cheng[2], and Jiqiang Ling[1, 3*]

Supplementary Data

Oligos. *rpsD* I199N (5'-T*G*T*G*TCCTCTCTTTGGTACTAAGCTTTACTTGGAGTAAAGCTCG ACGTTAAGGTGTTCGTTAATGTCCGCAGACAGATCAGAACGCTCCG -3'), rpsL K42N (5'-T*C*A*G*ACGAACACGGCATACTTTACGCAGCGCGGAGTTCGGTTTGTTTGGAGTGGTAGT ATATACACGAGTACATACGCCACGTTTTTGCG -3'), rpsL K42N revert (5'- T*C*A*G*ACGAA CACGGCATACTTTACGCAGCGCGGAGTTCGGTTTTTTAGGAGTGGTAGTATATACACGAGT ACATACGCCACGTTTTTGCG -3')

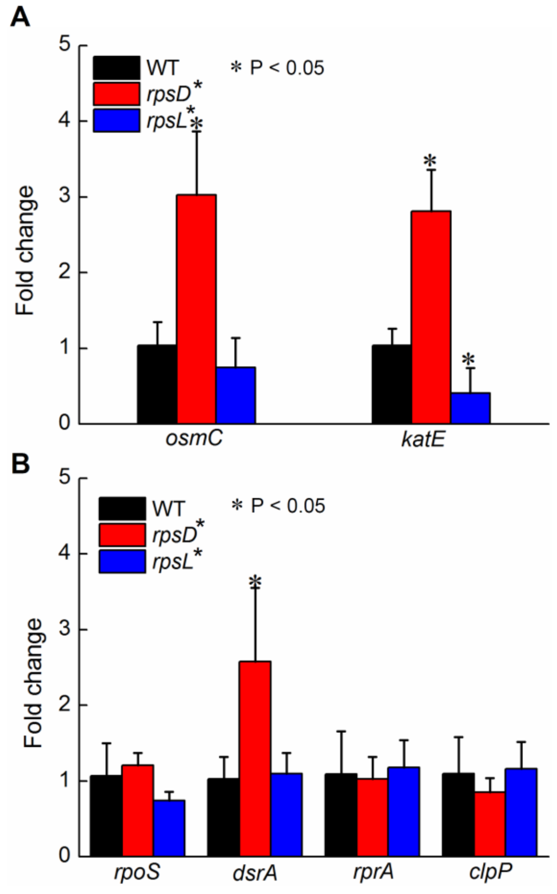

Figure S1. Gene expression levels determined by qRT-PCR. The wild-type (WT), error-prone *rpsD**, and error-restrictive *rpsL** strains were grown to mid-log phase in LB, harvested, and extracted for total RNA. qRT-PCR was used to determine the mRNA levels of indicated genes. The error bars represent standard deviations (n ≥ 3).

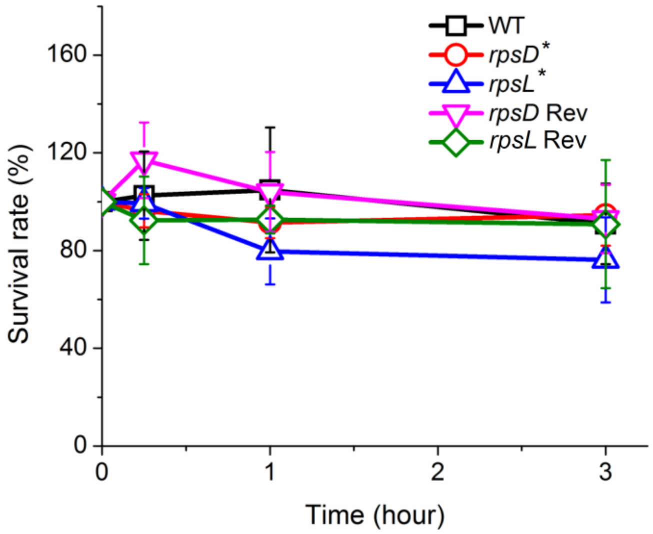

Figure S2. Survival of *E. coli* strains without H_2O_2. *E. coli* strains were grown to mid-log phase and tested for survival without H_2O_2. The error bars represent standard deviations (n ≥ 3). Rev, revertant.

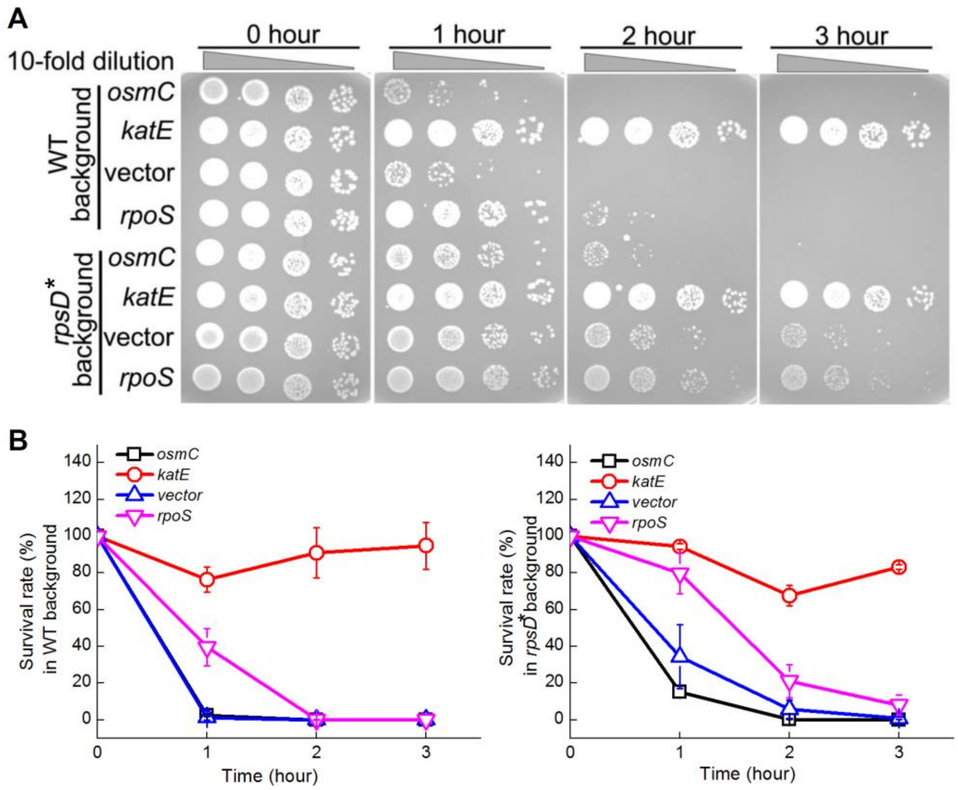

Figure S3. Survival or the WT and *rpsD* strains overexpressing KatE, OsmC, and RpoS in the presence of H_2O_2. The WT and *rpsD** strain carrying empty vector or overexpression plasmids (controlled by *lac* promoter) were grown in the presence of 0.1 mM IPTG to mid-log phase, and tested for survival in the presence of 5 mM H_2O_2. (A) Representative survival figures. (B) Quantitation of survival rates. The error bars represent standard deviations (n ≥ 3).

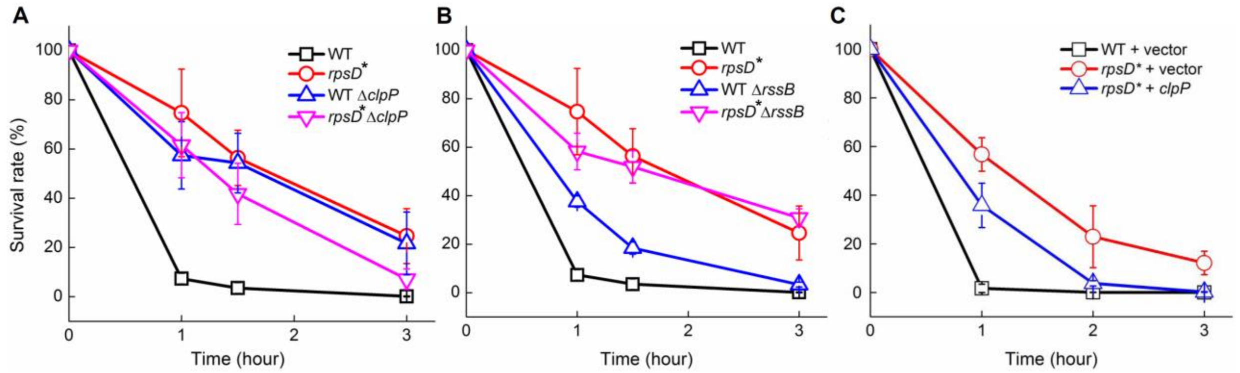

Figure S4. Role of ClpP and RssB in protection against H_2O_2. Cells were grown to mid-log phase and tested for survival in the presence of 5 mM H_2O_2. (A) Deleting ClpP, which degrades RpoS, protected the WT against H_2O_2. (B) Deleting the adaptor protein RssB, which recruits RpoS to ClpP, also increased tolerance to H_2O_2. The error bars represent standard deviations ($n \geq 3$). (C) Overexpressing ClpP partially suppresses the improved H_2O_2 tolerance from the *rpsD** mutation.

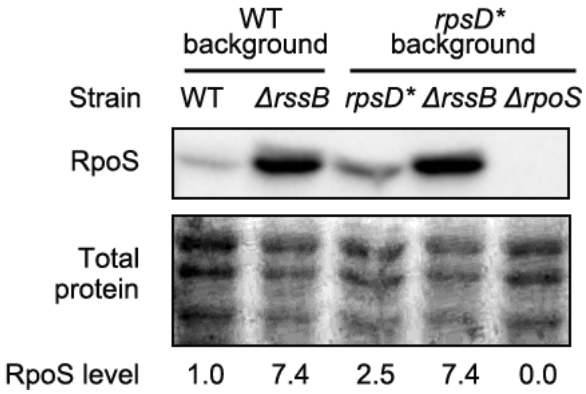

Figure S5. Deleting *rssB* increases RpoS protein level. *E. coli* strains were grown to mid-log phase, and Western blot against RpoS (top panel) and Ponceau staining of total proteins (bottom panel) were performed. Equal amount of total proteins were loaded in each lane. The figures are representatives of three repeats.

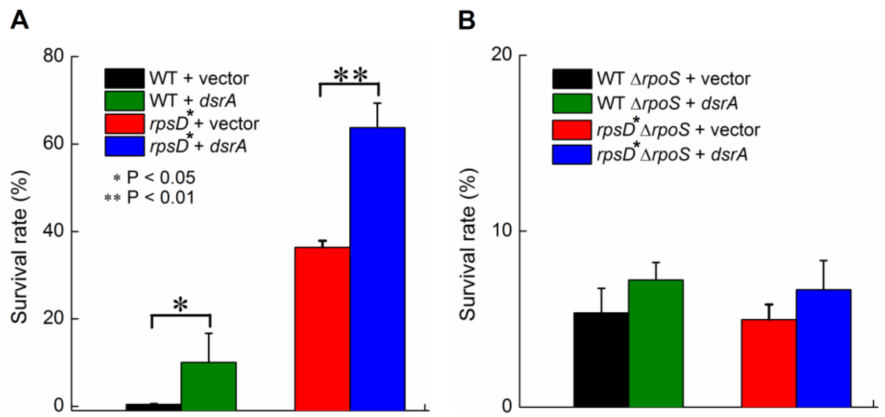

Figure S6. Overexpression of DsrA protects against H_2O_2 in a manner dependent on RpoS. (A) WT background. Cells at mid-log phase were treated with 5 mM H_2O_2 for 3 hours before plating. (B) *rpoS* deletion background. Cells at mid-log phase were treated with 5 mM H_2O_2 for 0.5 hour before plating. The error bars represent standard deviations (n ≥ 3).

Table S1. RNA sequencing results of the WT, *rpsD*, and *rpsL* strains. Small RNAs, including tRNAs, were removed during sample preparation for RNA sequencing. Quantitation of small RNA levels in the RNA sequencing result was therefore not accurate.

Table S2. Table S Several oxidative response factors with known functions

Name	Product	Role	Regulation
katE	Catalase HPII	Scavenging of H_2O_2	RpoS response (1)
osmC	Lipoyl-dependent Cys-based peroxidase	Reduction of organic hydroperoxide	RpoS response (2)
katG	Catalase G	Scavenging of H_2O_2	OxyR response (3)
ahpCF	Alkyl hydroperoxide reductase	Scavenging of H_2O_2	OxyR response (3)

References

1. Ivanova, A., Miller, C., Glinsky, G. and Eisenstark, A. (1994) Role of rpoS (katF) in oxyR - independent regulation of hydroperoxidase I in Escherichia coli. *Molecular microbiology*, **12**, 571-578.
2. Conter, A., Gangneux, C., Suzanne, M. and Gutierrez, C. (2001) Survival of Escherichia coli during long-term starvation: effects of aeration, NaCl, and the rpoS and osmC gene products. *Research in microbiology*, **152**, 17-26.
3. Imlay, J.A. (2008) Cellular defenses against superoxide and hydrogen peroxide. *Annu. Rev. Biochem.*, **77**, 755-776.
